# Review of the Newly Developed, Mobile Optical Sensors for Real-Time Measurement of the Atmospheric Particulate Matter Concentration

**DOI:** 10.3390/mi12040416

**Published:** 2021-04-09

**Authors:** Sama Molaie, Paolo Lino

**Affiliations:** Department of Electrical and Information Engineering, Polytechnic University of Bari, 70125 Bari, Italy; paolo.lino@poliba.it

**Keywords:** mobile monitoring, air quality, particulate matter, air pollution, low-cost particle device, optical particle sensors, scattering techniques, image processing methods

## Abstract

Due to the adverse effects on human health and the environment, air quality monitoring, specifically particulate matter (PM), has received increased attention over the last decades. Most of the research and policy actions have been focused on decreasing PM pollution and the development of air monitoring technologies, resulting in a decline of total ambient PM concentrations. For these reasons, there is a continually increasing interest in mobile, low-cost, and real-time PM detection instruments in both indoor and outdoor environments. However, to the best of the authors’ knowledge, there is no recent literature review on the development of newly designed mobile and compact optical PM sensors. With this aim, this paper gives an overview of the most recent advances in mobile optical particle counters (OPCs) and camera-based optical devices to detect particulate matter concentration. Firstly, the paper summarizes the particulate matter effects on human health and the environment and introduces the major particulate matter classes, sources, and characteristics. Then, it illustrates the different theories, detection methods, and operating principles of the newly developed portable optical sensors based on light scattering (OPCs) and image processing (camera-based sensors), including their advantages and disadvantages. A discussion concludes the review by comparing different novel optical devices in terms of structures, parameters, and detection sensitivity.

## 1. Introduction

Air pollution is one of the fundamental problems of the modern world as it has adverse effects on human health and the environment [1,2]. According to the World Health Organization (WHO), air pollution, as the world’s most considerable single environmental health risk, contributes to 4.3 million and 3.7 million deaths from indoor and outdoor environments each year, respectively [3]. Particulate matter (PM), carbon monoxide, ozone, nitrogen dioxide, sulfur dioxide, and lead are six primary hazardous air pollutants [4] emitted from indoor and outdoor sources [5,6,7] with deleterious impacts on public health and the ecosystem. Due to the increase of population in the urban environment, particulate matter detection as a critical indicator of air pollutions has received considerable attention [8]. There is an exciting background of efforts used to recognize the harmful effects of PM air pollution. Numerous toxicological and controlled-exposure studies have demonstrated the severe impacts of particulate matter on the global earth climate, human mental health, and visibility over the past decades [9,10].

Generally, particulate matter, as a complex mixture of very tiny solid particles and liquid droplets, is composed of different chemicals. These suspended particles of various sources [11,12] vary in composition depending on spatial [13] and temporal scales [14].

There are various air quality monitoring techniques and high-time resolution instruments used mainly in fixed stations (e.g., concentration particle counter (CPC), scanning mobility particle sizer (SMPS), and continuous aerosol mass monitor (CAMM)) [15]. Apart from their advantages, these stationary instruments are usually expensive, large, complex, and need a time-consuming detection process. However, there is always a concern regarding the limited number of monitoring stations for high-sensitivity air quality monitoring. In recent decades, there has been a growing interest in mobile monitoring of particulate matter detection [16,17]. However, the detection accuracy of mobile devices is not comparable to stationary, professional instruments. Still, the compact structure among real-time detection is the main advantage of using these devices [18].

In order to meet the growing demand for real-time and portable air quality monitoring, various new solid-state optical sensor technologies and compact PM detection instruments have been recently proposed. The optical PM portable instruments fall into two primary categories, i.e., scattering-based optical counters (OPC) and camera-based optical sensors. The working principle of OPCs [19,20,21] is based on scattering theories such as the Mie or Ryleigh theory. The amplitude of the scattered light from particles can be measured by the use of a single particle photodetector. The scattered intensity is proportional to the size of any individual particles in the sampling chamber [22]. The intensity of light scattered by particles of diameter r for a laser light wavelength of λ follows different scattering theories. For a small sphere diameter r≪λ, the intensity is proportional to $r^{6}$. However, by increasing the particle size to r≫λ, the scattered light intensity becomes proportional to the squared particle radius $r^{2}$. The advantage of these devices is the application range from low concentration (e.g., clean room) to high concentration industrial aerosols (e.g., industrial filter testing) [23,24]. However, there is a limitation of higher number concentration for OPCs, due to particle coincidence for detecting micron and submicron size particles defined by manufacturers [25,26].

The detection of the second category of camera-based optical sensors relies on the analysis of pictures captured by a 2D matrix sensor (camera) by image processing techniques. Inside the inlet chamber, the backlight illuminates the particles, and the camera records the images. Due to the use of a 2D camera sensor, the probability of particle coincidence is not a limitation at high concentration. The sensor can also extract information on multiple particles at once.

This paper aims to analyze and categorize recently established scattering-based optical particle counters and camera-based optical particle devices. The authors have made a first attempt to give an overview of mobile optical particle counters for air quality detection in [27]. However, the referred paper strictly focuses on optical particle counters based on scattering techniques and a single detector and does not cover low-cost optical PM sensors. Thus, to the best of the author’s knowledge, this is the first review considering newly designed optical particles counters based both on scattering and novel camera-based optical sensors, involving a critical discussion on their advantages and disadvantages. The paper is organized as follows. Section 2 describes a general introduction to the particulate matter effects and sources. It also provides a summary of standard requirements for controlling air quality. Section 3 reports the overview of the optical PM sensors. Section 4 presents the general OPC device principle. Section 5 compares the most recent developed optical particle counters, while Section 6 describes the newly designed optical PM sensors based on image processing. Section 7 focuses on a comprehensive comparison between different air quality sensors. Finally, Section 8 draws some conclusions.

## 2. Particulate Matter Characteristics and Legislation Emission

### 2.1. Particulate Matter Effects on Health and the Environment

In particular, growing evidence implicates the increase of primary chronic diseases and risk of premature mortality by exposure to PM pollutants [28]. Long-term exposure to air pollution has been associated with a rise in disease rates among older people, from non-accidental cardiovascular and respiratory diseases to lung cancer mortality [28,29]. However, epidemiological studies and evidence from the last decades have shown the lesser adverse effects of PM exposure on cerebrovascular diseases [8]. Different studies have also consistently shown an association between exposure to PM air pollution during pregnancy and not only the risk of infant mortality and reduced birth weight [30], but also preterm birth (PTB) and stillbirth [31].

Besides, a wide-ranging relationship exists between particulate matter exposure and depression or suicide [32]. There is also a growing awareness of the potential for adverse neurological effects of particulate matter on cognitive impairment and hospitalization for headaches [33,34]. Particulate matter can enter the blood and even reach the brain [35] or lead to inheritable mutation [36]. Although increased day-to-day risks from PM exposure remains to be mediated for any individual, the cost of global healthcare maintenance is overgrowing.

**Figure 1:**
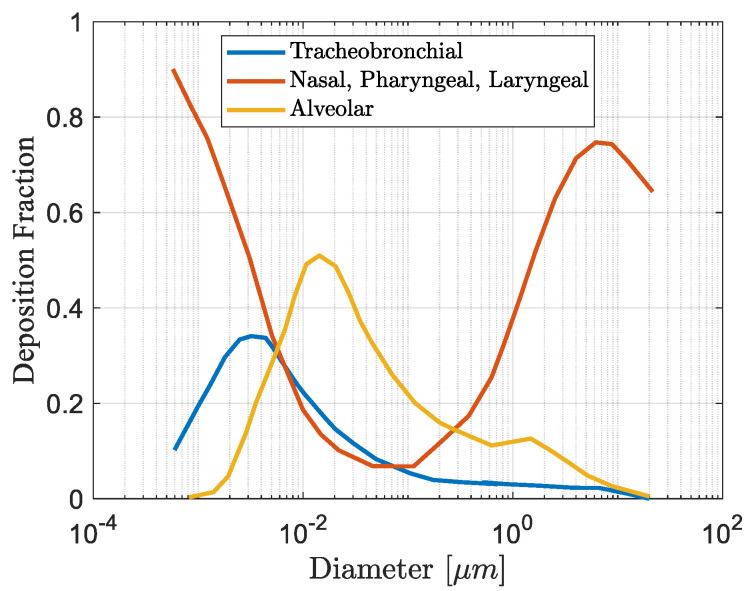
The representation of the deposition fraction of the particulate matter vs. particulate matter diameter in different parts of the airway system based on the International Commission on Radiological Protection (ICRP) model.

Particulate matter with different aerodynamic diameters can be deposited in nasal airways [37]. Figure 1 shows the impact of particulate matter deposition on the airway system during nasal breathing, according to the International Commission on Radiological Protection (1994) model of fractional deposition of inhaled particles [38,39]. Based on the ICRP model, various parts of the human respiratory tract (such as the nasopharyngeal) can be affected by particulate matter emissions in the ambient air.

Several studies have consistently shown a dose-dependent association between PM exposure and human morbidity in which any decrease of PM from the environment limits the outbreak of the diseases. At the same time, more research is required to recognize PM chemical composition’s effect on human health and mortality.

One more concern regarding the influence of PM pollution on climate change and visibility includes both the direct and indirect effects. Direct effects include scattering and absorption of the sunlight by PM in the atmosphere, whereas indirect effects are associated with cloud cover changing by particles serving as cloud condensation nuclei [40]. Besides, decreasing the atmosphere visibility due to light scattering by PM causes a noticeable reduction of the light intensity contrast between a distant object and background sky [40,41].

### 2.2. Particulate Matter Sources and Regulation Requirements

Particulate matter originating from stationary or mobile sources is classified into two categories. Primary particles are generated directly from specific emission sources, i.e., transportation sources (e.g., road traffic and aircraft) [42,43], industrial sources or operating power plants (e.g., industrial processes for different materials), natural sources (e.g., forest fires or dust storms), and household activities (e.g., cooking and heating) [44]. Secondary particles result from chemical reactions between pollutants (e.g., sulphur oxides and nitrogen oxides in the atmosphere). In this framework, significant efforts have been made to decrease air pollution and eliminate the hazardous outcomes of atmospheric PM on human health and well-being. Different organizations provide the regulations for threshold pollutant concentration to control the air quality in the urban environment.

The European emission standards also established the limitation of heavy-duty and light-duty vehicle exhaust emissions. This limitation led to the optimization of the combustion engine and improved the after-treatment system [45] by manufacturers. There are also different guidelines, such as the U.S Environmental Protection Agency (EPA) regulation [46], the European Union (EU) standards [47], the World Health Organization (WHO) guidelines [48], and the Chinese National Ambient Air Quality Standards (GB 3095-2012) [49], that set mass-based ambient air quality standards for particulate matter diameter of 2.5 micrometers and smaller (PM2.5), and PM of diameter 10 micrometers and smaller (PM10). Table 1 shows the maximum permitted PM mass provided by the EPA, the WHO, the EU, and the Chinese 2012 NAAQS.

## 3. Overview of the Mobile Optical PM Sensors

Due to the increasing concern for controlling particulate matter, the demand for low-cost and portable monitoring devices is continuously growing day by day. Various small and compact particulate matter sensors exist for monitoring indoor and outdoor air quality offered by different companies. However, recent significant advances in low-cost mobile sensors make it necessary to review some recently developed optical PM devices.

The sensors can analyze the data either by scattering techniques or image processing methods. Generally, optical sensors determine the particles’ size and number concentration and convert them to particle mass concentration. With this aim, it is assumed that particles are spherical and characterized by uniform density. These assumptions are not necessarily valid for airborne particles in an urban environment, although they are standard approximations [50]. The particle mass is obtained by multiplying the particle density and the calculated spherical volume of the particles. Table 2 shows the candidate sensors organized by their principle of operations and their general characteristics.

## 4. Optical Particle Counter Principle

### 4.1. Light Scattering Theory

The first mathematical model of light scattering was developed in the 19th century. Light scattering refers to the process where an electromagnetic (EM) wave (e.g., an incident light wave) encounters an obstacle (e.g., a particle), and the light photon redirects into a new trajectory. Generally, photon light can deviate from any heterogeneity in the medium that has been illuminated by incident light [22,51].

**Figure 2:**
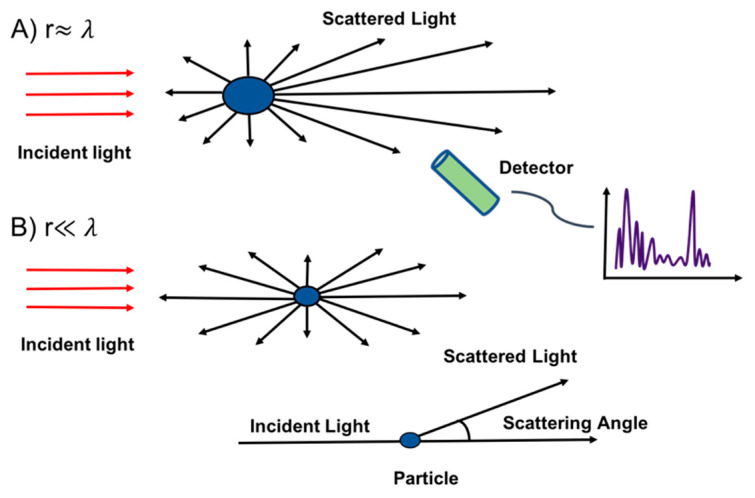
Light scattering principle. (**A**) the Mie theory for large particles, (**B**) the Ryleigh theory for small scale particles.

Elastic scattering occurs when the wavelength of the scattered light is the same as the incident light. Elastic scattering of electromagnetic radiation by particles can be described by the Mie theory, the Ryleigh theory, and geometry optics [52]. It is possible to define the size parameter x according to the following formula [52]:

(1) x=2πr/λ,

where r is the particle length (radius), and λ is the wavelength of the incidence light. The Mie theory is usually valid when the size of the particle is comparable to the wavelength of light, i.e., x=1. Generally, when the size of the particle is much smaller than the light wavelength, i.e., x ≪1, Mie theory converges to Rayleigh scattering. For x ≫1, Mie scattering theory gradually converts to geometric optics with the increase of particle size. Figure 2 represents the light scattering process when each particle in the medium is exposed to the incident light.

When the monochromatic plane wave illuminates a spherical particle, the intensity of the light scattering of a single particle based on the Mie theory is described in the following equation

(2) $$I_{S}=\frac{\lambda^{2}I_{0}}{4\pi R^{2}}\left({\left|S_{1}\right|}^{2}\;\sin^{2}\varphi +{\left|S_{2}\right|}^{2}\cos^{2}\varphi \right),$$

where, *λ* is the wavelength of light source, $I_{0}$ is the light intensity, *R* is the distance between observation point and particle, $S_{1}\;$ and $S_{2}\;$ are the amplitude function linked to a scattering angle of θ (the angle between the direction of light beam propagation and scattered light). In addition, φ stands for the angle between the vibration plane of incident light and scattering surface.

The scattered light intensity $I_{S}$ from small, single particle with diameter of d and reflective index of n , based on the Ryleigh theory is given by the following formula:

$$I_{S}=I_{0}\frac{1+\cos^{2}\theta }{2R^{2}}\;{\left(\frac{2\pi }{\lambda }\right)}^{4}\;{\left(\frac{n^{2}-1}{n^{2}+2}\right)}^{2}{\left(\frac{d}{2}\right)}^{6}.$$

The intensity of scattered light at any scattering angle is proportional to $r^{6}$.

### 4.2. Optical Particle Counters Based on the Scattering Theory

**Figure 3:**
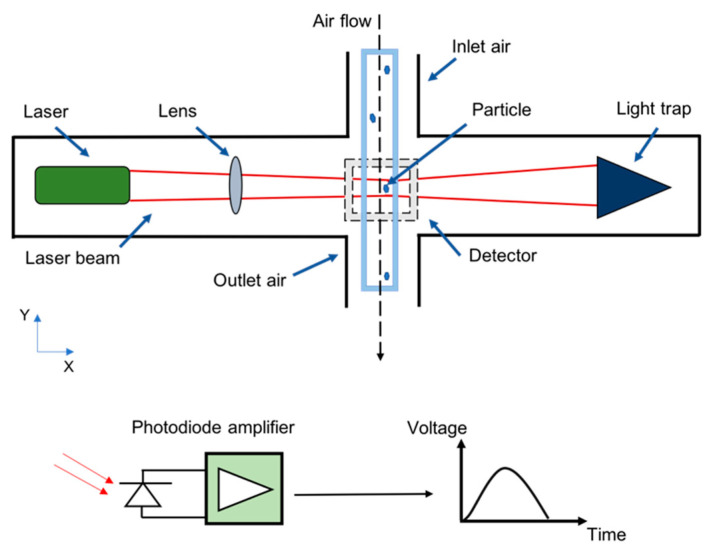
General structure of the optical particle counter.

Single-particle light scattering is widely exploited to detect particulate matter concentration in the urban environment. For example, commercial optical particle counters such as OPC–N3 (Alpha Sense Company, New York, NY, USA) [53,54], GP2Y1026AU0F [55], and the GRIMM model 1.107 [56], are based on this scattering principle. The overall structure of the OPC consists of a dark chamber, light source (e.g., light-emitting diode (LED) or laser), lenses, detector, light trap and duct inlet air. The sampled air flows inside the optical chamber using the miniaturized sampling nozzle. Inside the sensing volume, each particle illuminated by the laser scatters the light in all directions. A simple photodiode can be placed orthogonal to the laser light to detect the scattered light’s intensity. Then, the resulting signal is amplified using a pre-amplifier in the control circuit and converted into a digital signal that can be suitably processed. Usually, a horn shaped light trap (the opposite of the laser light) is adopted to avoid entering the stray light into the detection system. Based on the scattering Mie theory, each particle’s scattered light received by the photodetector generates a signal peak (see Figure 2) proportional to the particle’s size. Polystyrene latexes are commonly used to calibrate the optical particle counters [25,57]. Figure 3 shows the overall structure of the optical particle counter.

### 4.3. Coincidence in Optical Particle Counters

The introduction of the first OPC device dates back to the 1940s [58], and the successive improvements are strictly related to the development of lasers technology [59]. So far, a substantial improvement of the detection efficiency has been obtained thanks to the high intensity of the laser beam. Nowadays, OPC devices are widely used for the detection of particulate mass and particulate number concentration. However, to decrease the hazardous effects of air pollution and control the air quality, high performance and accurate OPC devices are requested. With this aim, several kinds of research and studies are focused on the principle errors of the optical particle counters, such as coincidence error [60].

One of the main errors in OPCs detection is the so-called “coincidence error”, which occurs due to the simultaneous presence of particles in the observation volume. Generally, the presence of more than one particle in observation volume can decrease the accuracy of the measurement. In fact, the size distribution can shift to a larger value as two particles or more count as one particle inside the observation volume [61]. Therefore the measured particle number concentration could be lower than the real one [62]. Generally, the upper number concentration of the optical particle counter is limited by the coincidence error [60]. To avoid the coincidence error, the presence of one particle in the observation volume is necessary. Considering the volume of observation, $V_{obs}$ and single particle assumption, maximum particle concentration can be defined as $C_{max}=1/V_{obs}$ $\left(\mathrm{particle}/m^{3}\right)$. The previous formula shows that there always exists a limit to the maximum concentration depending on the observation volume size. Besides, it is assumed that $\;c\leq c_{max}$ holds in the presence of one particle in the observation volume. Decreasing the observation volume is one of the solutions to reduce the coincidence probability [63]. However, the main problem is the difficulty in building a miniaturized inlet duct by manufacturers.

The adoption of a 2D matrix sensor instead of a single-pixel detector [64], providing an image of the detection volume’s interior, enables a precise counting of the number of particles. Accurate single particle detection is achieved when all particles in the observation volume are imaged at once.

## 5. Optical Particle Counters Based on Scattering Techniques

Light scattering-based sensors are almost universally used as compact particle detection systems. There are various sensors used daily by users for monitoring the air quality from indoor to outdoor monitoring. Among the portable instruments, low-cost and compact optical particle counters can determine particulate aerosol characterization, including mass and number concentration. Recently, researchers proposed new miniaturized and simplified scattering-based OPCs (in terms of detectors, light sources, and lenses) to enhance detection accuracy. This section describes different structures of the newly developed optical particle counters and compares their properties.

### 5.1. Smartphone-Based OPC

**Figure 4:**
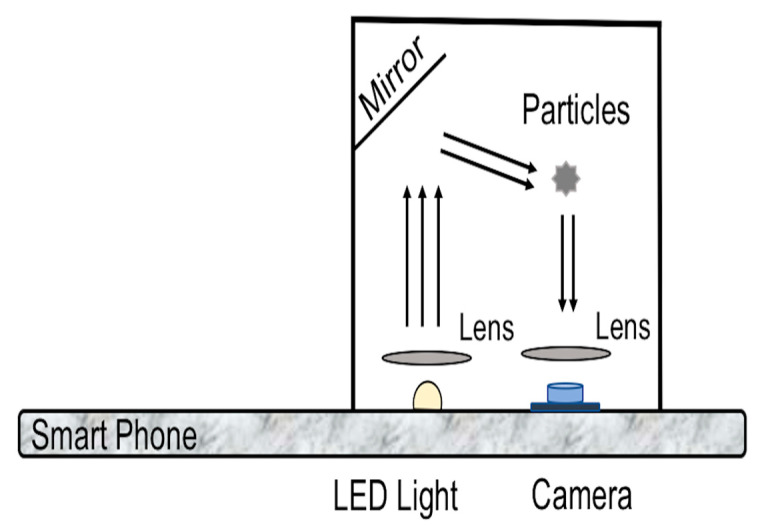
The third-generation design of the optical sensor.

Combining a miniaturized and low-cost optical dust sensor with a smartphone seems a promising approach to avoid any external and complex platforms and electronics for transmitting the measurement data [65]. The Sharp Gp2y1010AU0F ( Sharp Corporation, Sakai, Japan )belongs to the first generation of these dust sensors. The particles are illuminated by the LED flash of the smartphone, which is coupled with an optical fiber [66]. The phone camera, differently from a single-pixel photodetector, takes a series of images of particles. Then, the obtained images analysis gives the intensity of the scattered light. The device is able to measure down to a concentration level of 10 $\mathrm{mg}/m^{3}\;$ due to insufficient light intensity and low coupling efficiency of the LED to the optical fiber [66]. In the second generation (a.k.a. Mobile Dust), the introduction of semi-spherical lenses guaranteed a more intense and effective scattered light [67]. Inadequate light intensity and lack of details on the particle number in the observation volume led to the third generation (a.k.a. Fein Phone) of smartphone-based OPCs (Figure 4). In this case, a mirror is used instead of the optical fiber, and the 2D camera detects the scattered light from the particles after passing from a single lens (using a magnifier-based method). Besides, the use of a single large magnifying lens has a significant benefit in detecting the single scatter traces of particles instead of a sum signal [64]. Another feature of this compact sensor is that it can be easily mounted and removed from the smartphone. Table 3 gives a complete overview of the three generations of smartphones-based OPC.

**Figure 5:**
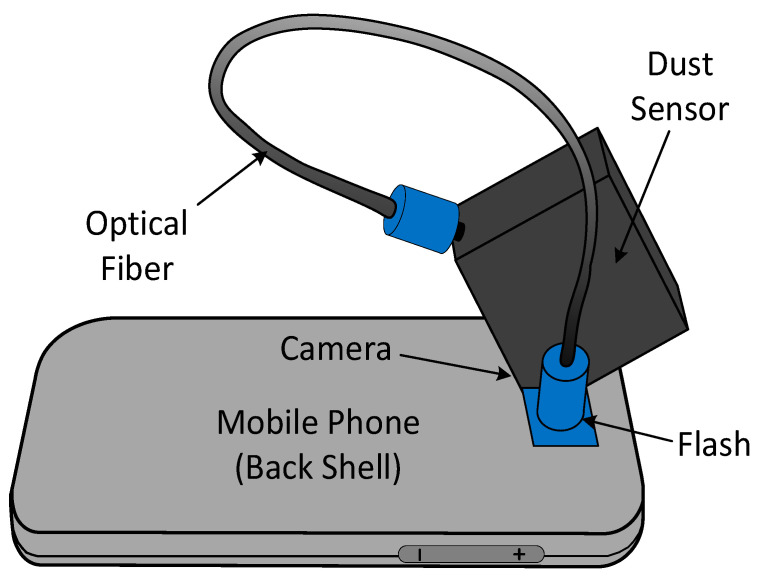
The total schematic of the active OPC. Adapted from Ref. [66].

It is possible to classify the smartphone-based optical particle counters into passive and active types, respectively. The passive version design directly depends on the phone characteristics, which affect the final configuration of the detection system. One major drawback of the passive version is the diffused LED light of the smartphone, which decreases the detection system accuracy. Conversely, the main advantages are the sensor’s low cost and the opportunity to implement the detection analysis directly on the phone software. There is also no extra cost for using the microcontroller or the headphone jack to transmit the data [62]. The active version is based on the use of an external LED independently from the phone model (Figure 5). However, the LED and micro-fan supplies require an external power source (e.g., extra batteries, solar panels, and phone audio interface). In addition to hardware design, adequate software technology can reduce user interface errors during the detection process. Table 4 gives a general comparison between the active and passive design of optical PM sensors.

### 5.2. Optical Particle Counter Based on the Parabolic Collector (Sub-Micron Particle Counter) CPC-Based OPC

Insufficient scattered light received by the photodiodes affects the accuracy of the particle counter in detecting sub-micron particles. A 90° detection angle applied to most commercial OPCs is not sufficient for this purpose. Besides, reliable sensors with a broad size range detection are expensive and need regular calibration (such as the TSI AM520).

**Figure 6:**
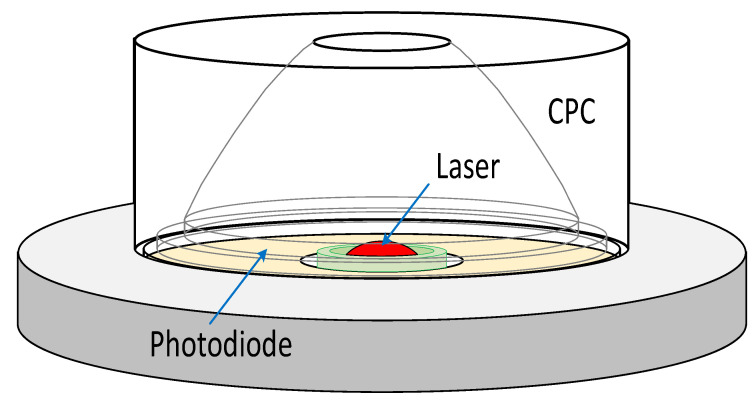
The schematic of the particle counter based on CPC. Adapted with permission from Ref. [68]. © 2021 IEEE.

A novel CPC-based OPC, collecting the scattering light from wide angles, can increase the intensity of the light received by the detector and obtains higher sensitivity [68]. Figure 6 shows a complete scheme of the OPC with a cylinder shape of 11 mm (height) × 25 mm (diameter). A laser diode light interacts with particles and scatters inside the sensing chamber. The scattered beam is firstly collected by a customized mirror based on a complicated parabolic collector (CPC). Then, the scattered light is detected by a silicon photodiode array.

In comparison to the two other developed sensors (SENSOR-90 based on 90° scattering and SENSOR-BACK based on back scattering show 37.1 $\mu g/m^{3}$ and 11.0 $\mu g/m^{3}$ resolutions respectively), the CPC-based sensor detection shows better performance with a higher resolution of about 7.9 $\mu g/m^{3}$. Moreover, a linear relationship is held between the particle concentration and the output voltage of light scattered intensity for CPC-based sensor, SENSOR-90 and SENSOR-BACK from the laboratory measurement results. The device can also be used for air quality monitoring because the sensor resolution is lower than the 24-h limitation of the WHO standard for PM2.5 (25 $\mu g/m^{3}$) [69].

A numerical analysis based on the Mie theory [70,71] shows that the scattering light focuses on the forward direction by increasing the particle size. Therefore, by selecting an appropriate CPC angle using a numerical calculation, the photodetector can easily detect the light scattered by submicron particles. The proposed CPC within a small port of 4.5 mm and a length of 9 mm use an acceptance angle of 20° degree, to accurately detect the submicron particles. Decreasing the background noise by applying the effective electromagnetic shielding for the pre-amplifier, polishing the CPC, and using a high stability laser driving circuit, increases the sensitivity of the CPC-based sensor [68].

### 5.3. Silicon-Based OPC (MEMS PM Monitor)

A recently developed low consumption, light scattering-based sensor on a miniaturized scale of 15 mm × 10 mm × 1 mm can be used in cellphone and smartwatch-based systems [72]. The sensing unit consists of two silicon chips: the bottom is the actual sensing unit, including a laser and a detector, while the top wafer contains the inlet and outlet airflow channels for the particulate matter. Both are connected to an external control circuitry using metal pads in the silicon sub-mount corners.

**Figure 7:**
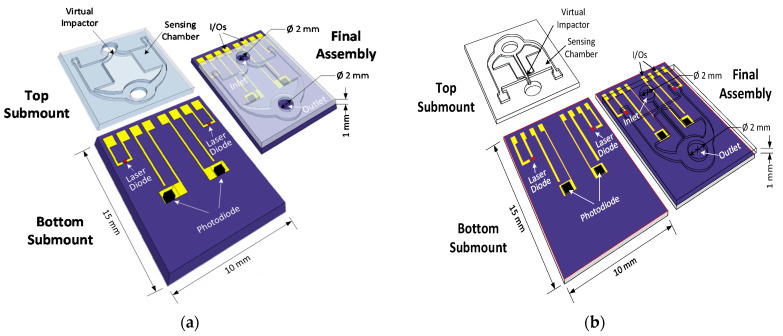
The schematic of the designed particle sensor; top submount is flipped over bottom submount, so as to overlap surfaces, as sketched in the final assembly. (**a**) The sensing unit of the second layout of silicon-based OPC. Adapted with permission from ref. [72]. © 2021 Elsevier; (**b**) The schematics of the third layout PM2.5 sensor. Adapted with permission from ref. [74]. © 2021 IEEE.

In a first layout [73], the silicon nitride anti-reflection layer (ARI) with an 80 nm thickness is deposited on the inner chamber surface to minimize the reflection. Alternatively, in a second version [72] (Figure 7a), it used a robust material of multilayer ARI (TiO_2_ SiO_2_) layer. This material reduces the reflectivity of silicon (which is above 30% for visible light) and avoids ARI thickness deviation dependency. A micro-pump draws out the air to the outlet by a flow rate of 20 mL/min in negative pressure. Experimental tests showed a measurement accuracy of less than 10 $\mu g/m^{3}$ without any particle selection [72].

Optimizing the dimension of the sensing chamber can reduce light reflection and increase the detector output. For example, a reduction of 0.5 mm on the long side of the chamber from 3.5 mm can increase the detector output to 100%.

In a third prototype [74], a particle selector (virtual impactor (VI)) is integrated into the top sub-mount and after the air inlet. The airflow rate remains very low and helps to sustain the low power consumption of the sensor. Compared to previous versions [72], several factors contribute to better performance in terms of mass sensing accuracy. The airflow passes the air inlet and goes through the impactor by the use of a jet. The small particles enter the sensor chamber while the large particles exit from the outlet channel. Since particles of various sizes follow different scattering theories, the selection of the fine particles by the integrated VI filter can enhance the system detection accuracy. A collimating channel inside the PM sensor is used to suppress the direct laser light received by the detector and to increase the sensor accuracy. On the other hand, reflected light from the chamber surface could increase the system noise. Figure 7b represents the third layout of the PM sensor. Experimental results indicate a high detection accuracy of 2.55 $\mu g/m^{3}$ for the system.

### 5.4. Fresnel Ring Lens-Based OPC and the Inertial Filtering

**Figure 8:**
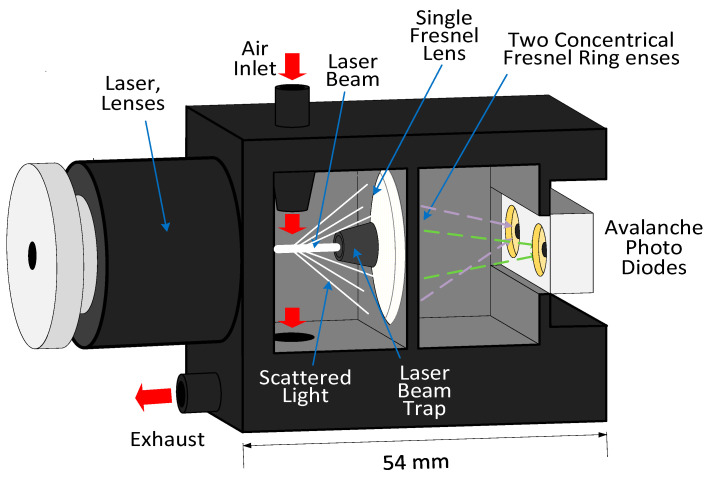
Fresnel ring lens-based OPC. Adapted with permission from Ref. [77]. © 2021 Optical Society of America.

Recently, a novel particle sensor based on an inertial filtering system and the collection of scattered light intensity at two solid angles with Fresnel lenses was proposed [75]. The sensor design consists of two subsystems. The first one includes an impactor filter (IF) attached to the inlet optical sensor to select the desired particles according to their mean aerodynamic diameters. The IFs collect the particle of the size of interest on an impaction plate (e.g., Marple-type impactor filter) [76] depending on their inertia. The second subsystem includes the light scattering-based sensor. The laser light illuminates the airborne particulate matters entering the inlet sensor, then two avalanche photodiodes detect the scattered light intensity. The computing system finally derives the particle size distribution. The sensing chamber includes Fresnel lenses instead of the spherical lenses to collect the particles’ scattered light [77]. Figure 8 shows the overall layout of the sensor detection system.

In the sensing chamber, the particulate matter is directed near the laser beam waist using an inlet nozzle to increase the scattered light intensity. Three Fresnel ring lenses with large numerical aperture are located in the forward scattering direction and near the scattered light source to obtain further light fraction [78].

The first frontal Fresnel lens around the laser beam trap collimates the scattered light. Then, the two Fresnel ring lenses behind the primary lens concentrate the light scattered by the particles on the two avalanche photodiodes (APD). Finally, the particle size is deduced from the scattered light intensity at the two different known angles.

This optical particle counter can detect particle sizes ranging from 140 nm to several microns, with a high precision of 10 to 20% [77,78]. Detection results obtained on polystyrene latex and silica particles in a size range of 300 to 1600 nm were in good agreement with the theoretical calculation. It is expected that this could monitor the particle size and mass concentration in indoor and outdoor environments. In order to determine the particle density distribution of the multiple particle sizes, the particle size distribution (PSD) can be defined without inertial filtering and with IF connected to the optical particle counter. Filtering efficiency can be measured by difference of both measured particle size distribution. Finally, the actual density is obtained using the Stokes number equation. The particle mass concentration can be determined by multiplying the particle density and the defined spherical particle volume.

### 5.5. Drilled Lens-Based OPC

**Figure 9:**
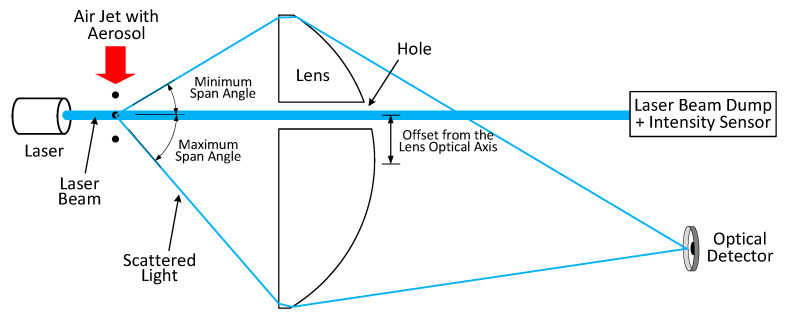
The layout of optical particle counter based on a drilled plastic lens. Adapted with permission from Ref. [79]. © 2021 American Association for Aerosol Research.

A simple high sensitive and low-cost optical particle counter based on the detection of the forward light scattering from small particles has been recently proposed in [79]. In the sensing chamber, a spherical imaging lens with a small hole drilled in front of the laser collects the forward scattered light and separates it from the laser beam (see Figure 9). An air jet containing the particles intersects the laser beam. The laser light ends in a dump after passing through a drilled hole in the lens, while the forward scattered light by particles is collected on the opposite side of the drilled hole. The scattered light intensity is detected by a photomultiplier to determine the size distribution of the particles with a known refractive index.

The main disadvantage of this system is that the numerical aperture of the lens restricts the angular range of the collected light. The hole diameter of the lens determines the lowest light collection angle. Experimental results performed on polystyrene latex particles indicates a detection range of 125 nm with high sensitivity [79]. A theoretical analysis shows that a lower size detection range of 100 nm can be obtained by reducing the background noise of the scattered light from the apertures and the beam dump. This low-cost sensor can be employed for balloon-borne and ground-based detection of PM. The estimated cost of a complete aerosol PM sensor is 2000 dollars.

## 6. Optical Particle Sensors Based on Image Processing Techniques

Over the past decade, air quality monitoring using optical particle sensors has gained growing attention [25]. The light scattering approach’s main disadvantage is the rising probability of coincidence at high densities, which sensibly reduces the detection accuracy of the sensor [80,81]. Newly designed optical particle devices based on image processing provide a compact and precise detection solution. Unlike one-dimensional scattering based methods, which determine the total light intensity of particles by a photodetector, the new devices exploit a 2D matrix sensor (camera) detecting all particles in the sample volume at once. The number or mass distribution can be calculated from the captured images by suitable image processing techniques.

### 6.1. Holographic Particle Counter

**Figure 10:**
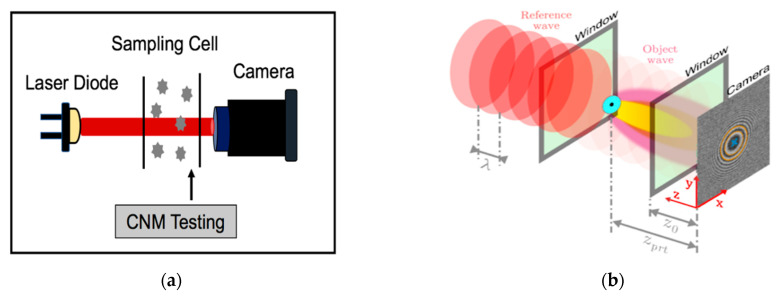
The layout of optical holographic particle counter; (**a**) holographic counting unit; (**b**) holographic principle and creation of a fringe pattern with a camera detection system.

A novel holographic particle system (Figure 10a) to detect the number concentration of micrometer-sized particles was recently described in [82,83,84]. As previously mentioned, the increasing probability of particle coincidence in higher density samples is an inevitable drawback of the widely used light scattering based instruments [60]. However, to detect every single particle, a complete screen of the sensing volume at once is required. This holographic detection system suggests using a 2D camera sensor instead of a single photodiode to obtain full 3D information on the observation volume. In further detail, it can provide information on the size, position, direction, and speed of the motion of the particles. However, this system’s main limitation is the overlapping of the fringe patterns created in the camera plan due to higher particle densities and the coincidence of particles along the light beam. To avoid an overlap of fringe patterns and to obtain detectable particles at higher concentrations, it is necessary to define an appropriate distance of the particle from the detector (z-component) with sufficient pixel resolution. The *z*-axis defines the size of the fringe pattern on the detection plane (Figure 10b). Preferably, a smaller depth of field (DOF) leads to a lower particle coincidence probability. It is worth noting that the proposed imaging device is also a zero-particle monitoring system due to the direct imaging of particles in the observation volume.

A novel particle imaging unit [84] including three necessary parts of the in-line holography model, aerosol particle model, and computational fluid dynamics simulation for generating a virtual hologram plane of multiple particles of different sizes, is presented. Figure 10 shows the working principle of the optical holographic counting setup [85], representing its main parts: the light source (laser) as a reference plane wave, a uniformly distributed particle sampling cell, and a 2D camera as a detection imaging system. Each particle in the unit flows into the sampling channel and is exposed to the 1 mW power diode laser beam. The particle diffracts the incident beam and creates a circular interference fringe pattern on the detection plane. The particle number concentration is extracted from the recorded images by means of pattern recognition techniques [86] based on the Hough Transform (HT) method, even in noisy images. The Hough Transform technique is a robust extraction method of digital image processing for detecting single geometrical shapes such as straight lines, circles, etc. [86].

This setup can easily be attached to the Condensation Nucleus Magnifier (CNM), which operates based on magnifying the small particles before exposure to the laser light. Therefore, the sensor is intended as a novel alternative to condensation particle counters (CPCs), which can operate in higher densities (>30.000 $\neq /{\mathrm{cm}}^{3}$). The system’s main advantage is that it does not require a narrow inlet for counting each grown particle in the observation volume, and it is independent of the speed and direction of the particles motion.

There is a good linear relationship between the particle counting rate of the particle imaging unit (PIU) and detected particle number concentration measured by the reference TSI-3775 particle counter below 600 ($\neq /{\mathrm{cm}}^{3}$). At higher concentrations, with the increasing probability of coinciding particles and overlapping of the fringe patterns in the detection plane, two coincidence correction approaches can be applied, i.e., the Dead Area Correction and the Lambert-W function. Both methods enable comparable results in the low concentration area. At higher concentration rates, the Dead Area Correction continues to underestimate, while the Lambert-W function considers the rising probability of multiple hidden particles inside the sampling volume [82]. The detection sensitivity of the proposed sensor is around 4.16 ($\neq /{\mathrm{cm}}^{3}$) in single-shot mode and can be improved by increasing the frame rate of the camera or the total sampling time.

### 6.2. Filter-Based Particle Sensor

**Figure 11:**
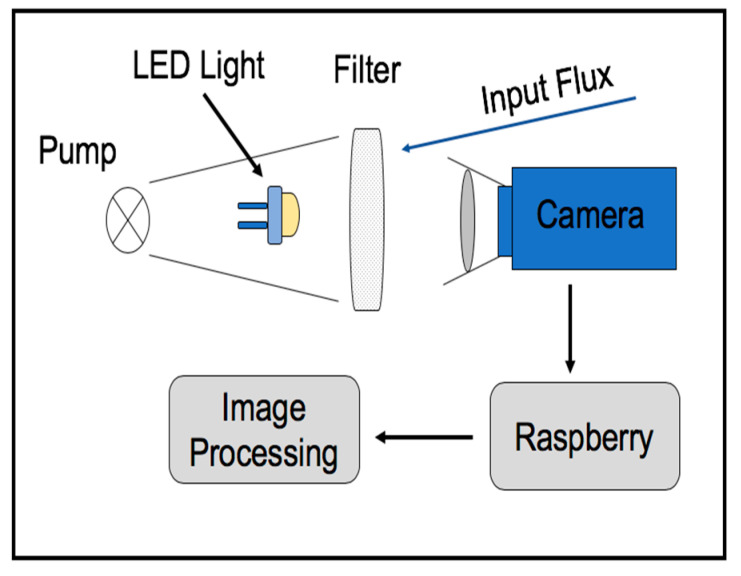
The structure of the filter-based sensing chamber.

Another low-cost camera-based sensor, using a glass-fiber filter for detecting the PM of sizes smaller than 2.5 μm in the urban environment, is proposed in [87,88]. The particulate matter transported by the airflow, which is regulated by a small constant-flow pump, enters the inlet sampling and passes through the filter (Figure 11). Two servo motors push the air through the filter to prevent any air leakage. A stepper motor can replace the saturated filter, if necessary. Inside the sensing chamber, a set of multi-wavelength LEDs illuminates the surface of the filter [89]. The front camera periodically takes photos to detect the filter’s captured particles, which appear as dark spots on the background light. Finally, the size distribution of the particulate matter is extracted from the captured images using a suitable image processing software. The image processing software is based on the Open Source Computer Vision (OpenCV) library (Intel Corporation, Santa Clara, CA, USA) and performs four processing phases to analyze the images [90]. Firstly, gaussian blur is applied to grayscale images to decrease the consequence of background filter fiber. Then, 2D kernel filtering enhances image sharpness. Finally, different particle sizes are extracted using a blob detection algorithm by a progressive threshold binary matching method.

It is worth mentioning that the transparency of the particles exposed to different LED wavelengths can easily change. Therefore, analyzing the captured photos in the same filter with different transparency gives useful information about particle composition.

A cheap digital camera is connected to a Raspberry PI Zero W(Raspberry Pi Foundation, Cambridge, England), which is adopted as the central controller of the system. It features a 32- bit 1 GHz ARM microprocessor (Arm Ltd., Cambridge, England) with 512 MB RAM, and operates in a Linux environment. In addition, several sampling nodes are connected with each other over some kilometers by using the low-cost and long-range transmission protocol of the LoRa wireless module RF-LORA-868-SO (RF solutions, Burgess Hill, West Sussex, England). The monitoring system is able to join the Wi-Fi network provided by RPI Zero to create a remote connection with availability to access the data.

In order to assess the efficiency of the proposed sensor, different PM concentration measurements were performed, and the results were also compared with a commercial light scattering-based monitoring system. The average concentration of PM10 detected by the filter mesh of 3 μm in a day was about 0.36 $\mu g/m^{3}$, which is comparable with commercial monitoring stations performance. This sensor is relatively cheap, with an approximate components cost of about 200 dollars. Improvements should contemplate the filter mesh size, power consumption, increasing the flow rate (using the pump), and decreasing the device size for the next generation of the system.

### 6.3. Dust Deposition-Based Particle Sensor

**Figure 12:**
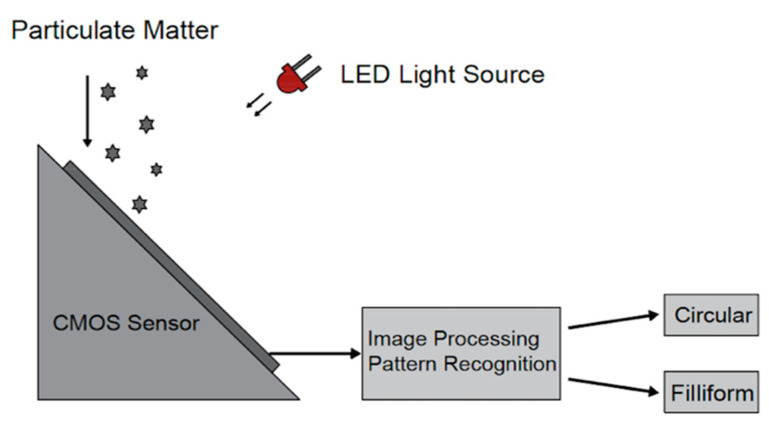
Dust sensor setup based on deposition on the CMOS camera.

**Figure 13:**
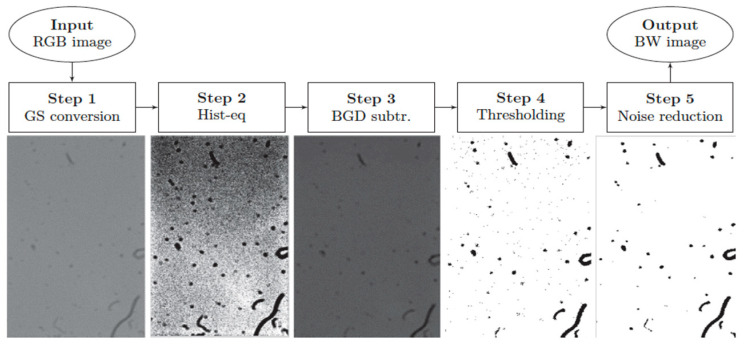
The preprocessing steps for detection of the particles. Adapted with permission from [92]. © 2021 Elsevier.

One more detection system based on a Complementary metal–oxide–semiconductor (CMOS) image sensor, able to determine the size and shape of the particles by means of image processing techniques, classification algorithms, and pattern recognition, is described in [91,92]. Figure 12 shows a schematic representation of this system. The particles directly deposit on the camera surface (without lenses), placed at a 45° angle towards the ground to limit the overlapping due to particle accumulation. A white LED light illuminates the sensor surface to record the high-contrast photos in various environmental conditions. The control software allows taking a picture periodically and saves it as an uncompressed JPEG. The image processing of the images captured by the camera is performed in two steps: a preprocessing algorithm reduces the noise or the residual flickering and enhances the contrast, then a pattern recognition and classification algorithm extracts the size and shape of the particles. The first step develops in five different phases (Figure 13). Firstly, the recorded images are converted to grayscale to reduce the computational requirements. Then, an histogram equalization (HE) enhances the contrast. A low pass filter eliminates the brightness gradient of the image background and removes it from the original image. Furthermore, the subtracted images convert to binary images based on the Otsu method [93]. Eventually, a median filter (with a square mask size of 3 pixels) removes any residual noise from the binarized image. Since the pixels of the images are proportional to the particle size, an increase of the covered pixel by the particle at captured photos can be compared. Therefore, the accumulated dust rate on the sensor surface can be obtained by reversing the image in white on black and calculating the percentage of dirt on the recorded image. In the second step of the process, the reliable pattern recognition algorithm based on the k-NN (k-Nearest neighbor) classifier is employed on the preprocessed images. This algorithm only uses the proper metric to assess the distance between the patterns and define the shape and size of the particles. Based on dust features, the classification algorithm is able to subdivide the captured images into six labels. The test results confirm the accuracy of higher than 90% for the experimental analysis.

It is possible to obtain the dust features and sources from geometrical and topological data of the particle size and shape. For instance, circular dust and filiform elements originate from particulate matter and fiber sources, respectively. This information can help governments to control dust pollution by appropriate preventive actions. Figure 12 shows the image acquisition system based on the deposition of the dust particles on the CMOS image sensor (The Microsoft HD-3000 imaging camera (Microsoft Corporation, Redmond, Washington, United States) obtains a pixel size of 3 μm [92]).

The described approach appears promising for detecting the dust deposition size and shape in a fixed indoor environment such as museums, surgery, or clean rooms. In order to validate the sensor, several tests were performed [91].

## 7. Comparison and Discussion

Table 5 and Table 6 summarize the specifications of the technological optical particle sensors for air quality monitoring. As it is evident, all sensors are amongst two main categories of light scattering and image processing techniques. A comparison between the different devices can be assessed with reference to two main aspects. The first one relates to the detectable particle size range for accurate measurement of a single particle and to the particle concentration. The second one regards the device’s dimension and miniaturization, cost, and other criteria related to consumer use [18]. With this aim, Table 5 reports the typical detection range of the PM based on functional operation, the different structures, and other limitations of the sensors. Table 6 shows the main components and dimensions of the newly designed portable instruments.

The system based on the Fresnel lens shows a lower detection size of 125 μm within the compact structure. However, using avalanche photodiodes and Fresnel lenses can raise the overall cost of the sensor. In the PM2.5 range, the landscape of miniatured devices is dominated by silicon-based OPC, used from home with smartphones and smartwatches. This sensor achieves an acceptable accuracy of 2.55 $\mu g/m^{3}$ and provides a quick response to any variation of the particle concentration.

The miniaturized smartphone-based OPC layout depends on the phone model, but the control of the detection system can be performed on any smartphone. However, the measurable particle size is limited to the inhalable coarse particle (PM (10–2.5)) range, mostly due to the insufficient LED flashlight of the smartphone. The main advantage of this sensor is its ultra-low cost and the capability to be attached and removed quickly from the phone.

Among them, the low-cost CPC-based sensor enhances the detection accuracy of sub-micron particles, providing a wide collection angle of the scattered light. The miniaturized sensor with a high measurement accuracy of about 7.9 $\mu g/m^{3}$ can easily monitor the air quality in the metal 3D printing workshop. However, the system performance should be improved by decreasing the noise from the metal CPC.

The simple drilled-lens OPC based on forward scattering and laser beam separation can monitor 125 nm particles with high accuracy. Although, since it is based on accurate optical components, the sensor is still bulky and costly and, thus, not appropriate for extensive mobile monitoring. The low cost and simple design COMS image sensor is able to determine detailed information on the shape and size of the particles and identify the dust origins. It is valuable to know that the sensor can efficiently be used in fixed indoor environments such as museums or cleanrooms. As it is evident, the mobile filter-based sensor using wide wireless connectivity seems to be a promising candidate for low-cost PM monitoring. However, the sensor still needs some improvements in terms of the sampling system, detection size range, and dimensions. Among the others, the holographic particle counter is a compelling alternative to the condensation particle counter (CPC) for determining the airborne particle number concentration lower than μm-rang. The sensor enables accurate measurement of the low particle number concentration and enables the determination of higher particle densities.

## 8. Conclusions

Due to the hazardous effects of particulate matter on human life and the environment, monitoring of air pollution and, more specifically, particulate matter concentration are among the leading social concerns. There are extensive researches and investigations on different stationary devices whose accuracy fulfills their respective standards.

However, due to the different PM sources and weather conditions, the PM concentration in the urban area is significantly different at a local scale [94]. In this framework, fixed stations always have the limitation of real-time monitoring of the spatial and temporal variability of PM. Several portable and reliable PM monitoring devices with different structures, principles, and platforms were recently developed to overcome this limitation. With the advent of mobile devices performing high temporal-spatial resolution and real-time detection, air quality monitoring approaches have gradually changed. At present, mobile optical sensors can monitor the classified components of PM (PM1, PM2.5, PM10), in terms of size, mass, and number concentration. However, mobile PM monitoring methods are much less known if compared to the classic air monitoring techniques. This review briefly summarizes the development and current status of the newly developed OPCs, and of camera-based optical sensors for measuring the mass, number, and size of the PM. The aim is to direct the interested reader to more in-depth studies. Both types of sensors are characterized by benefits and shortfalls, and the final choice depends on the purpose of monitoring and the available budget. The proposed techniques appear very promising for the development of PM air monitoring sensors. However, there are still some issues related to the particles’ detection size and accuracy. Since demands for portable sensors is considerably increasing, it is expected that more accurate devices based on new structures and processing methods for real-time characterization of PM will appear soon.

## Figures and Tables

**Table 1:** Air quality standards defended by the U.S Environmental Protection Agency, the World Health Organization, the European Union, and the Chinese 2012 National Ambient Air Quality Standards.

Air Quality Standards	PM2.5 (Annual Mean)	PM2.5 (24-h Mean)	PM10 (Annual Mean)	PM10 (24-h Mean)
EPA	15 ($\frac{\mu g}{m^{3}})$	35 ($\frac{\mu g}{m^{3}})$	-	150 ($\frac{\mu g}{m^{3}})$
WHO	10 ($\frac{\mu g}{m^{3}})$	25 ($\frac{\mu g}{m^{3}})$	20 ($\frac{\mu g}{m^{3}})$	50 ($\frac{\mu g}{m^{3}})$
EU	25 ($\frac{\mu g}{m^{3}})$	-	40 ($\frac{\mu g}{m^{3}})$	50 ($\frac{\mu g}{m^{3}})$
2012 NAAQS	35 ($\frac{\mu g}{m^{3}})$	75 ($\frac{\mu g}{m^{3}})$	70 ($\frac{\mu g}{m^{3}})$	150 ($\frac{\mu g}{m^{3}})$

**Table 2:** Two main categories of low-cost mobile optical particle detection sensors.

Mobile, Low-Cost Optical Particle Sensors			
Principle	Light Scattering Method (Mie Theory, Raleigh Theory)		Image Processing Methods
Detection system	Photodiode	Camera	Camera
Examples	Commercial OPC	Smartphone based OPC	Holography sensors

**Table 3:** Comparison of different PM sensor generation.

OPC Based on Smart Phone	First Generation Sensor/Second Generation Sensor	Third Generation Sensor
Type of smart phone used in research	HTC smart phone(HTC Corporation, New Taipei, Taiwan)/Google Nexus 4 (LG Electronics, Seoul, South Korea) Android 2.3.3	Galaxy S6 (Samsung Electronics, Seoul, South Korea) Android 7
System design	Using optical fiber/Semi-spherical lens using optical fiber	Mirror-based using camera LED
Detection size	Coarse particle	Fine particle
Method of processing	Based on sum light scatter of the particles	Based on scatter traces of the particles (magnifier-based approach)
Processing approach	Poisson Particle Detection (PPD) algorithm	Combing the PPD algorithm and Contour Detection Particle Counting(CDPC) algorithm
Detection	Online detection	The data can analyses for further check of algorithm parameter
Camera position	In focal point of lens	Far from focal point of the lens
Dimension	Bulky and large dimension	Small dimension and easily install

**Table 4:** Comparing the active and the passive design of the smartphone-based OPC.

Type of the Smartphone-Based OPC	Passive Design	Active Design
Structure	Depends on phone	Independent of phone
System hardware	Should be adopted for different phones	Use for wide range of smart phones
System control	Software implementation in phone	External control
Cost	Ultra-low cost	low cost
Light source	Insufficient intensity using flash of the phone	Sufficient light using external LED

**Table 5:** Summary of the main newly developed mobile optical sensors reporting information on instrumentation, measured parameters, and resolution.

Kind of Sensor	Working Principle	Detected PM Size Ranges	Minimum Resolution	Detection System	Reference System
CPC-based OPC	Light scattering	PM1	7.9$\;\mu g/m^{3}$	Mass concentration	Dust track DRX TSI18533
Silicon-based OPC	Light scattering	PM2.5	2.55 $\mu g/m^{3}$	Mass concentration	OPC 1.109 GRIMM
Drilled lens-basedOPC	Light scattering	PM2.5 (PM above 140 nm)	_	Mass concentration	Single particle soot photometer
Fresnel ring lens-based OPC	Light scattering	PM2.5 (PM above 125 nm)	_	Size detection/mass concentration	Grimm SMPS + C5.403
Camera-based OPC	Light scattering +signal processing algorithm	PM10–2.5	_	Size detection/ mass concentration	SMPS/TSI APS
Holographic particle counter	Holography+ Image processing	PM above 3–4 μm after condensation	Singleframe: 4.16 ($\neq /{\mathrm{cm}}^{3}$) 20 fps: 0.208 ($\neq /{\mathrm{cm}}^{3}$)	Number Concentration	TSI-3775
Camera filter-based sensor	Image processing	PM above 3 μm	_	Size detection/ mass concentration	Commercial particulate monitoring station
CMOS sensor	Image processing	PM above 3 μm	_	Size detection/shape	_

**Table 6:** Comparison of the components of the novel sensors.

Kind of Sensor	Light Detection	Detector	External Lens	Dimension
CPC-based OPC	Laser diode (635 nm)	Five red-enhanced Si photodiode	CPC/20 °C acceptance angle	Miniaturized (11 mm × 25 mm)
Silicon-based OPC	Laser diode (650 nm)	Silicon PIN Photodiode	Collimating channel	Miniaturized (15 mm × 10 mm× 1 mm)
Drilled lens-based OPC	Laser diode (405 nm)	Photomultiplier	Aspheric lens with hole	Small (length: 300 mm)
Fresnel ring lens-based OPC	Laser diode (450 nm)	Two avalanche photodiodes	Fresnel ring lenses	Miniaturized (Measurement chamber: 54 mm)
Camera-based OPC	Smartphone flash LED	Smartphone camera	Large magnification lens	Miniaturized (Depends on phone model)
Holographic particle counter	Laser diode (635 nm)	UI-3082SE-M Camera IDS (COMS sensor)	_	Small (Without CNM)
Camera filter-based sensor	3RGBLED 1 IR LED 1 UVLED	RPI NOIR Camera V2	Macro Lens 15×	Small
CMOS sensor	White LED	LifeCam HD-3000 (COMS sensor)	Without lens segment of camera	Small

## Data Availability

Exclude this statement.
